# Supplementary material for: Genomic signals of ecogeographic adaptation in a wild relative are associated with improved wheat performance under drought stress
Source: Genome Biol. 2025 Feb 21;26:35. doi: 10.1186/s13059-025-03500-1 (PMC11844086; doi:10.1186/s13059-025-03500-1)
Supplement: Supplementary file 2 — Additional File 2: Figs S1-S4. Fig. S1. The effect of geographic, climatic and bioclimatic variables on the SNP diversity in Ae. tauschii population. Fig. S2. Relationship between the number of days to heading (DTH) and yield, and the genomic loci associated with days to heading of Ae. tauschii introgression lines. Fig. S3. Manhattan plots showing CAAs on chromosomes 1D, 2D, 6D and 7D significantly associated with yield and yield component traits in the Ae. tauschii introgression population. Fig. S4. Importance of canopy temperature and yield component traits for yield prediction based on random forest model in Ae. tauschii-wheat introgression population. [file 13059_2025_3500_MOESM2_ESM.docx]

**Fig. S1.** The effect of geographic, climatic and bioclimatic variables on the SNP diversity in *Ae. tauschii* population. **A.** Stack barplot showing the ancestry proportion shared amongst the *Ae. tauschii* lines at K=4. **B.** Principal component plot for *Ae. tauschii* lines. The filled circles represent the 21 lines used to generate the introgression population. **C.** Redundancy analysis biplot showing the effect of both geography and bioclimatic variables on *Ae. tauschii* diversity. Sub-lineage 2 West (L2W) and sub-lineage 2 East (L2E) are *Ae. tauschii* ssp. *strangulata* whereas sub-lineage 1 East (L1E) and sub-lineage 1 West (L1W) are *Ae. tauschii* ssp. *tauschii*. **D.** Redundancy analysis biplot based on geographic variables.

**Fig. S2.** Relationship between the number of days to heading (DTH) and yield, and the genomic loci associated with days to heading of *Ae. tauschii* introgression lines. **A.** Deviation in the number of days to heading (DDTH) for the ILs from the controls (parents and checks) mean days to heading. **B.** Manhattan plot showing significant associations for heading date on 2DS and 7DS at a threshold FDR 0.05 indicated by the red line. **C-E.** Yield (bushels per acre) for the ILs in the tails of DDTH distribution under non-irrigated conditions at Colby 2018, Colby 2019, and Ashland 2020, respectively. Asterisks (**) indicate significant difference (t-test, P = 0.034).

**Fig. S3.** Manhattan plots showing CAAs on chromosomes 1D, 2D, 6D and 7D significantly associated with yield and yield component traits in the *Ae. tauschii* introgression population. Where (A) is grain length, (B) is grain width, (C) is spikelet number per spike and (D) is grain yield. Panels A-C are based on BLUPs as phenotypes and CMLM model whereas D is based on spatial adjusted yield at Colby in 2019 under non-irrigated conditions using MLMM model. SNPs above the red line are significantly associated with traits after FDR correction at alpha value 0.05.

**Fig. S4.** Importance of canopy temperature and yield component traits for yield prediction based on random forest model in *Ae. tauschii*-Wheat introgression population.
