# Supplementary material for: Genomic signals of ecogeographic adaptation in a wild relative are associated with improved wheat performance under drought stress
Source: Genome Biol. 2025 Feb 21;26:35. doi: 10.1186/s13059-025-03500-1 (PMC11844086; doi:10.1186/s13059-025-03500-1)
Supplement: Supplementary file 8 — Additional File 8: Table S7. SNPs significantly associated with days to heading, yield and component traits in Ae. tasuchii introgression population. [file 13059_2025_3500_MOESM8_ESM.docx]

**Table S7.** SNPs significantly associated with days to heading, yield and component traits in *Ae. tasuchii* introgression population.

| **SNP** | **Chr** | **Position (RegSeq 2.0)** | **P.value** | **MAF** | **nobs** | **R^2 Model without SNP** | **R^2 Model with SNP** | **FDR_Adj_P-values** | **Effect** | **Model** | **Trait** |
| --- | --- | --- | --- | --- | --- | --- | --- | --- | --- | --- | --- |
| chr2D_22255525 | 2 | 22255525 | 3.09E-09 | 0.26513 | 347 | 0.171733 | 0.263167 | 1.75E-05 | -0.084335578 | CMLM | Grain length (GL) |
| chr2D_22165390 | 2 | 22165390 | 1.55E-08 | 0.35879 | 347 | 0.171733 | 0.254621 | 4.39E-05 | 0.074587399 | CMLM | Grain length (GL) |
| chr2D_22276164 | 2 | 22276164 | 3.72E-07 | 0.304035 | 347 | 0.171733 | 0.238032 | 0.000704599 | -0.071871267 | CMLM | Grain length (GL) |
| chr7D_8728438 | 7 | 8728438 | 2.00E-06 | 0.279539 | 347 | 0.171733 | 0.229439 | 0.002838756 | 0.057702447 | CMLM | Grain length (GL) |
| chr2D_61772585 | 2 | 61772585 | 2.30E-09 | 0.253602 | 347 | 0.02615 | 0.135516 | 1.30E-05 | 0.029718751 | CMLM | Grain width (GW) |
| chr6D_462184586 | 6 | 462184586 | 7.11E-07 | 0.332853 | 347 | 0.02615 | 0.100202 | 0.002016761 | -0.023186001 | CMLM | Grain width (GW) |
| chr6D_462203291 | 6 | 462203291 | 2.62E-06 | 0.324207 | 347 | 0.02615 | 0.092403 | 0.004947538 | 0.022248565 | CMLM | Grain width (GW) |
| chr1D_432307193 | 1 | 432307193 | 1.73E-05 | 0.273775 | 347 | 0.02615 | 0.081253 | 0.016373083 | 0.022991084 | CMLM | Grain width (GW) |
| chr1D_432367341 | 1 | 432367341 | 1.73E-05 | 0.273775 | 347 | 0.02615 | 0.081253 | 0.016373083 | 0.022991084 | CMLM | Grain width (GW) |
| chr1D_432386924 | 1 | 432386924 | 1.73E-05 | 0.273775 | 347 | 0.02615 | 0.081253 | 0.016373083 | 0.022991084 | CMLM | Grain width (GW) |
| chr7D_13654672 | 7 | 13654672 | 4.45E-06 | 0.317771 | 332 | 0.168401 | 0.224891 | 0.025237531 | -0.242082536 | CMLM | Spikelet number per spike (SNS) |
| chr2D_31779118 | 2 | 31779118 | 8.93E-06 | 0.164157 | 332 | 0.168401 | 0.221202 | 0.025333185 | 0.259578291 | CMLM | Spikelet number per spike (SNS) |
| chr2D_22165390 | 2 | 22165390 | 3.21E-07 | 0.342988 | 328 | NA | NA | 0.001824437 | NA | MLMM | Yield |
| chr7D_55774657 | 7 | 55774657 | 8.00E-09 | 0.4121212 | 330 | NA | NA | 4.54E-05 | NA | MLMM | Days to heading (DTH) |
| chr7D_35345332 | 7 | 35345332 | 2.87E-07 | 0.075757 | 330 | NA | NA | 0.000815731 | NA | MLMM | Days to heading (DTH) |
| chr2D_61772585 | 2 | 61772585 | 3.76E-06 | 0.231818 | 330 | NA | NA | 0.007113905 | NA | MLMM | Days to heading (DTH) |
